# Successful Treatment of Lower Limb Complex Regional Pain Syndrome following Three Weeks of Hyperbaric Oxygen Therapy

**DOI:** 10.1155/2016/3458371

**Published:** 2016-03-30

**Authors:** Rita Katznelson, Shira C. Segal, Hance Clarke

**Affiliations:** ^1^Department of Anesthesia and Pain Management, Toronto General Hospital, University Health Network, Toronto, ON, Canada M5G 2C4; ^2^Division of Hyperbaric Medicine, Toronto General Hospital, Toronto, ON, Canada M5G 2C4

## Abstract

Hyperbaric oxygen therapy (HBOT) is a treatment that delivers 100% oxygen at increased atmospheric pressures. The efficacy of HBOT for treating pain has been described in various animal pain models and may have clinical efficacy in the treatment of human chronic pain syndromes. We present our experience with posttraumatic Complex Regional Pain Syndrome (CRPS) type 2 in a patient who underwent 15 sessions of HBOT. A 41-year-old male with one-year history of CRPS of left foot followed by left ankle fracture demonstrated less pain, decreased swelling, less allodynia, and improvement in skin color and range of motion of the lower limb after 3 weeks of HBOT. Patient was back to work for the first time in over a year. HBOT may be considered as a valuable therapeutic tool in the treatment of long-standing CRPS.

## 1. Introduction

Mechanisms of Complex Regional Pain Syndrome (CRPS) are poorly understood. Tissue hypoxia and inflammation can play an important role in the pathophysiology of this potentially debilitating condition [1–3]. We present the case of patient (G.G.) with CRPS type 2 of his lower leg that responded to a 3-week course of hyperbaric oxygen therapy. On February 21, 2014, G.G. slipped on ice in a parking lot while leaving work for the day and sustained a Weber B left ankle fracture. On February 23, 2014, he underwent an open reduction internal fixation of his ankle. His postoperative course was complicated with a cellulitis infection treated with antibiotics. Intense acute pain developed in his left ankle and foot immediately after the injury and the pain was very difficult to control postoperatively. G.G. developed persistent pain, which he described as having intermittent shooting qualities and severe electric shock episodes. It was accompanied with swelling, skin discoloration, allodynia, and temperature changes leading to diagnosis of CRPS of the left foot in April 2014. The computed tomography of the left ankle 6 months after surgery demonstrated union of the lateral malleolar fracture. Ultrasound of the left ankle at the same time was unremarkable. The patient was engaged in a rehabilitation program that focused on pain management, desensitization techniques, range of motion exercises, balance, gait retraining, and endurance training. G.G. was under the care of a chronic pain physician and his medication regimen included pregabalin 75 mg twice daily, acetaminophen and NSAIDs as needed, multivitamins, calcium, magnesium, and glucosamine. He experienced some relief in his symptoms by December 2014.

Approximately 1 year following his ankle injury, on February 5, 2015, G.G. underwent removal of his plateau in an attempt to alleviate his pain. Unfortunately, his symptoms such as pain, swelling, and allodynia intensified and he presented to our clinic with hopes that hyperbaric oxygen therapy (HBOT) could potentially improve his symptoms.

On March 17, 2015 (13 months since initial injury, 6 weeks since last surgery), prior to commencing HBOT, G.G. complained of a constant, dull, aching pain with intermittent shooting sensations. His numeric rating scale (NRS) pain score was 7-8/10 at its worst and 6/10 on average. Walking, standing, and sitting exacerbated the pain, and the pain was most intense by the evening. Additionally, allodynia around the lateral malleolus made everyday activities such as putting on socks and shoes quite difficult. The patient also reported weakness in his left lower leg, swelling around the ankle, and purple discoloration after prolonged standing or physiotherapy.

Physical examination revealed a purple, discolored, edematous area over the left fibula into the lateral malleolus, engorgement of the left ankle veins, atrophy of the left gastrocnemius muscle, and decreased skin temperature in the left foot. There were tenderness and sensitivity to touch below the lateral aspect of the left knee. Tinel's sign was positive in the distribution of the left superficial peroneal nerve with sharpness and tingling into the left first three digits. There were decreased left ankle range of motion, an impaired tolerance to plantar flexion endurance of the left foot and ankle, and a decreased walking distance by 6-minute walk test for age related norms.

## 2. Methods

Written Informed consent was obtained from the patient for publication of this case report. The patient is a 41-year-old man with CRPS of the left leg as a result of a work related injury. He received fifteen 90-minute HBOT sessions at 2.4 ATA, 1 session per day, 5 days per week for 3 weeks. The Brief Pain Inventory (BPI) and the Hospital Anxiety and Depression Scale (HADS) were administered at baseline and at completion of the treatment. The BPI asks respondents to rate their worst, least, average, and current pain on a 10-point scale (0 = no pain, 10 = pain as bad as you can imagine), followed by a rating of how much their pain interferes with everyday functioning (e.g., “walking ability,” “normal work,” and “mood”) [4]. HADS defines mood and anxiety as 0–7 = normal, 8–10 = borderline abnormal, and 11–21 = abnormal [5]. Pain scores were recorded hourly on a numerical rating with a scale of 0 = no pain at all, 5 = moderate pain, and 10 = worst pain possible.

## 3. Results

### 3.1. Pain Intensity

A pain diary was kept for the duration of the treatment, where hourly pain scores were collected. Prior to HBOT treatments G.G.'s baseline pain was 7/10. His average pain was 5.1 ± 0.73 on day 1 of the treatment, 4.5 ± 0.52 on day 7, 3.0 ± 0 on day 14, and 3.2 ± 0.41 at the end of treatment.

### 3.2. Brief Pain Inventory (BPI)

G.G. reported a 30% reduction in pain interference from baseline for general activity, 57% for mood, 44% for walking ability, 50% for normal work, 29% for relations with other people, and 30% for enjoyment of life.

### 3.3. Hospital Anxiety and Depression Score (HADS)

Before starting treatment, the patient scored 9 on the depression scale and 6 on the anxiety scale of the HADS. After treatment, his postscores were 6 on the depression scale and 4 on the anxiety scale, demonstrating an improvement in the patient's mood immediately after HBOT.

In summary after 3 weeks of 15 HBOT treatment sessions, the patient reported a progressive improvement in his symptoms. He had lower pain scores. The swelling and purple discoloration of his ankle were both reduced. At the end of HBOT treatment, his pain had reduced to 3.32/10 (mild range), and he reported being able to tolerate longer standing and walking without pain exacerbation. In addition, the area of allodynia around the scar and lateral malleolus almost disappeared. Tinel's sign in the distribution of the left superficial peroneal nerve became negative. The range of motion in the left ankle improved (dorsiflexion increased by 7°) and demonstrated 20% improvement in the 6-minute walk test. The patient tolerated hyperbaric oxygen treatments well with no complications.

### 3.4. 1-Month Follow-Up

G.G. was seen in follow-up 1 month following the completion of the HBOT treatments and his benefits were maintained. His pain was lower at 2/10, the discolored area of skin had returned to its normal color, and the reductions in swelling and allodynia had been maintained. Most impressively, G.G. had made a complete return to work and his regular duties as a fire fighter. Furthermore, he had started jogging—an activity he would not have been able to tolerate before HBOT.

### 3.5. 6-Month Follow-Up

G.G. was contacted by phone 6 months after the completion of the HBOT. He reported intermittent pain in his left ankle with the maximum intensity being 3/10 on NRS. He does not take any medications. He continues to work full time as a firefighter. He jogs short distances several times a week.

Taking these pre/postdifferences into account as well as the self-reported and objective improvements in appearance and functioning, this patient has demonstrated benefits from HBOT.

## 4. Discussion

This case report demonstrates a positive effect of HBOT in a patient with a one-year history of lower limb CRPS.

Hyperbaric oxygen therapy is a medical treatment defined as an intermittent inhalation of 100% oxygen in a hyperbaric chamber at a pressure higher than 1 absolute atmosphere (1 ATA = 760 mmHg, the normal atmospheric pressure at sea level). It is indicated for the treatment of decompression illness, gas embolism, delayed radiation injury, refractory osteomyelitis, necrotizing infections, compromised grafts and flaps, complex wounds, carbon monoxide poisoning, sudden neurosensory hearing loss, acute retinal artery occlusion, severe burns, and acute severe anemia. It has not been approved by FDA or Health Canada for treatment of CRPS or other chronic pain conditions. HBOT is a safe and reliable treatment with very few contraindications and side effects. The absolute contraindication is untreated pneumothorax. Common side effects are mild and reversible and include middle ear barotrauma, sinus pain, and myopia. Central nervous system toxicity is very rare and does not cause permanent damage. Pulmonary toxicity is dose related and can appear as dry cough, chest tightening, and temporary impairment of pulmonary function. With careful patient selection, education, and following treatment protocols, side effects and complications are largely preventable.

The therapeutic effects of HBOT are based on a supraphysiologic increase in the amount of dissolved oxygen carried by the blood. This increase allows oxygenation of ischemic areas with compromised circulation. HBOT activates oxidant-antioxidant mechanisms via an endothelial nitric oxide (NO) pathway, which plays a key role [6] in stimulating secretion of growth factors such as vascular endothelial growth factor, hypoxia inducible factor-1, and stem cells. By activating signal transduction cascades, HBOT has been shown to mediate tissue healing and improve postischemic and inflammatory injuries [7]. Furthermore, HBOT may cause an immediate and prolonged analgesic effect which is initiated and maintained by NO and NO-dependent release of endogenous opioids [8–10].

There is a growing body of evidence that HBOT decreases pain in different acute and chronic pain conditions [11]. Previous animal studies have highlighted the analgesic effect caused by HBOT, in nociceptive, inflammatory, and neuropathic pain models [10, 12, 13]. HBOT has been found to decrease mechanical hyperalgesia and inflammation in a rodent model. The antinociceptive effect was apparent immediately following HBOT and persisted up to 5 h posttreatment [12]. Inflamed tissues have increased cellular activity and consume more oxygen than noninflamed tissues. It is postulated that the improvements found after HBOT are related to the increased oxygen supply provided to the inflamed cells. Hyperoxygenation increases ATP production for energy related cellular functions and prevents anaerobic metabolism and tissue acidosis which has been associated with an increase in pain behaviours in animal models. Using a neuropathic pain model of L5 ligation and chronic constrictive injury of the sciatic nerve in rats, Thompson and colleagues demonstrated that 2 weeks of HBOT significantly improved pain levels and recovery during therapy and posttreatment [14]. The positive effect of HBOT on allodynia and hyperalgesia is postulated to be related to inhibition of endoneuronal TNF-*α* production [15].

In human trials, HBOT improved pain scores and range of motion in patients with idiopathic femoral head necrosis [16] and attenuated pelvic pain in females with interstitial cystitis [17]. HBOT had rapid onset and a long lasting effect in patients with idiopathic trigeminal neuralgia [18]. It was found to be an effective treatment in cluster headaches and migraine [19] and fibromyalgia [20, 21]. The potential benefits of HBOT in the treatment of CPRS were initially reported in a small double-blind, randomized, placebo-controlled study at a military hospital in Turkey on 71 patients with six-week history of traumatic CRPS of the upper extremity [22]. The treatment group received fifteen 90-minute HBOT sessions at 2.4 ATA, while the control group received fifteen 90-minute sessions in the hyperbaric chamber with normal air. The CRPS patients that received HBOT demonstrated significantly lower pain scores and improvement in edema and range of motion after the 15th treatment and at 45 days posttreatment (*p* < 0.001). The authors concluded that HBOT was an effective method for decreasing pain and swelling and increasing wrist range of motion in patients with new onset posttraumatic CRPS. The possible mechanisms explaining therapeutic effects of HBOT in CRPS could be related to tissue oxygenation, restoration of aerobic metabolism, correction of hypoxia, acidosis, and modulating NO activity and oxidative stress. This case is another supporting piece of evidence with respect to the above study.

It has been suggested that CRPS is related to dysfunction of the central and autonomic nervous systems [23–25], inflammation [26], immune system dysfunction [27, 28], and microvascular pathology leading to tissue hypoxia and ischemia [3, 29]. A recent study, which examined the effects of HBOT on patients with fibromyalgia, demonstrated that HBOT rectifies abnormal brain activity related to pain processing. HBOT decreased hyperactivity and blood flow in posterior brain regions and elevated activity and blood flow in the prefrontal cortex [21]. It is possible that similar mechanisms may have mediated the improvement in the CRPS symptoms seen by G.G.

G.G. presented with long-standing CRPS resistant to medical therapy and on disability. His symptoms flared up after a repeated surgical intervention aimed at pain reduction, which added an inflammatory component in addition to neuroplasticity changes. Following a 3-week course of HBOT therapy, G.G. had a significant reduction in pain intensity, allodynia, vasomotor symptoms, and pain disability which enabled improved function and return to full-time work.

## 5. Conclusion

HBOT may be effective in the treatment of chronic CRPS. Further randomized controlled trials in patients with long-standing CPRS should be conducted in order to define the role of HBOT in the treatment of this potentially devastating condition.

## Additional Points

Hyperbaric oxygen therapy (HBOT) is a treatment that delivers 100% oxygen at increased atmospheric pressures. We present our experience with posttraumatic Complex Regional Pain Syndrome (CRPS) in a patient who underwent 15 sessions of HBOT. After three weeks of HBOT, he had reduced pain scores, decreased swelling, less allodynia, and improvements in range of motion of the lower limb. Patient was back to work for the first time in over a year. HBOT may be considered as a valuable therapeutic tool in the treatment of long-standing CRPS.
